# Diametric Comparison between the Thoracodorsal Vessel and Deep Inferior Epigastric Vessel in Breast Reconstruction

**DOI:** 10.1155/2020/6352939

**Published:** 2020-07-16

**Authors:** Jun Hyeok Kim, Ye Sol Kim, Suk-Ho Moon, Young Joon Jun, Jong Won Rhie, Deuk Young Oh

**Affiliations:** Department of Plastic & Reconstructive Surgery, College of Medicine, The Catholic University of Korea, Seoul, Republic of Korea

## Abstract

**Background:**

In microvascular anastomosis, size discrepancy is common and can increase thrombotic complications. If size differences can be predicted, then vessels of the appropriate size can be selected. This study documented the difference in diameter between the thoracodorsal (TD) vessel and deep inferior epigastric perforator (DIEP) pedicle in each patient who underwent breast reconstruction using free tissue transfer. *Patients and Methods*. This retrospective study included 32 anastomoses (27 breasts including five cases of supercharged anastomosis) of breast reconstruction with the free DIEP flap and TD recipient between August 2018 and June 2019. In the microscopic view, the caliber of the TD vessel, the largest branch to the serratus anterior muscle, the descending branch, the largest and the second largest branches to the latissimus dorsi muscle, and the DIEP pedicle were measured.

**Results:**

The diameter of the deep inferior epigastric artery was similar to that of the descending branch, and their anastomosing rate was 56.3%. The diameter of the deep inferior epigastric vein was similar to the branch to the serratus anterior muscle and the descending branch, and their anastomosing rates were 29.3% and 29.3%, respectively. All flaps were survived; however, in one case, a reoperation was needed to remove the hematoma, in which case fat necrosis occurred as the only complication.

**Conclusion:**

TD branches of similar size to the DIEP pedicle were prioritized in anastomosis. The descending branch and the branch to the serratus anterior muscle are expected to be good candidates as recipients in breast reconstruction with DIEP free flap. Moreover, supercharged anastomosis of DIEP pedicles can be achieved within TD branches.

## 1. Introduction

The deep inferior epigastric perforator (DIEP) flap is considered the gold standard for excellent results in breast reconstruction [1–3]. It looks natural and achieves the consistency of the original breast, resulting in high cosmesis and even less morbidity at the donor site [4–6].

Nonetheless, microvascular anastomosis is an essential component of free flap transfers [7]. Size discrepancy of vessels is a common issue with these techniques, and it increases the rate of flap compromise by thrombotic risk [8–10]. Although many methods have been tried to overcome size discrepancy [10–16], a small difference in caliber reduces the rate of flap failure. If the size difference could be predicted, selection of the appropriate recipient site would be facilitated.

The thoracodorsal (TD) vessels are reliable and obtainable recipients in microsurgical breast reconstruction and are preserved during mastectomy and dissection of axillary lymph nodes [17, 18]. Even the advantage of using TD vessel is that it is easy and quick to prepare as the recipient vessel [19].

The TD artery has a small, favorable diameter for microsurgical anastomoses [17]; then, it is generally matched and suitable as the recipient of DIEP, even the success rate of anastomosis is up to 99.1%, and its thrombotic rate is low as 2.8% [19]. However, this clinical report is only an empirical statement without diametric comparison. A cadaveric study reports that TD vessels have smaller size discrepancy with DIEP than the internal mammary (IM) and circumflex scapular vessels as the better recipient site [20]; however, it has the limitation to connect with the clinical meaning like complication rates of anastomoses.

The aim of this study was to document the difference in diameter between the TD vessel as a recipient and the DIEP pedicle in each patient who underwent microsurgical breast reconstruction. This information allows the appropriate recipient site of the TD vessels to be predicted and a supercharging site also to be selected before surgery. To the best of our knowledge, this is the first clinical study to compare the diameter of TD branches with that of the DIEP pedicle in patients undergoing microvascular breast reconstruction.

## 2. Patients and Methods

**Figure 1:**
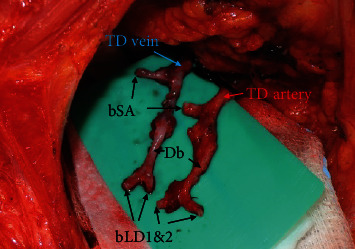
Prepared TD vessels under loupe magnification (×3.5). The surrounding soft tissue was removed from the vessels; its range extended from the bifurcation site with the circumflex scapular artery to the end of each branch; TD: thoracodorsal; bSA: the largest branch to the serratus anterior muscle; Db: the descending branch; bLD1: the largest branch to the latissimus dorsi muscle; bLD2: the second largest branch to the latissimus dorsi muscle; DIEP: deep inferior epigastric perforator.

**Figure 2:**
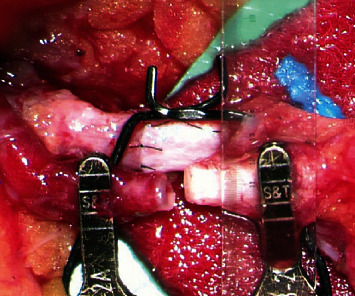
Use of the microscale ruler to measure vessel size in the microscopic view.

This retrospective study included 32 anastomoses (27 breasts including five cases of supercharged anastomosis) of the breast reconstruction with the free DIEP flap using the TD vessel as a recipient between August 2018 and June 2019. The average age of patients was 49.33 ± 7.27, and the average BMI was 23.16 ± 2.54. A microscale ruler (Crown Jun Microscale 0MR01, Kono Seisakusho, Japan) was used to measure the caliber of the TD vessel, the largest branch to the serratus anterior muscle, the descending branch, the largest branch to the latissimus dorsi muscle, the second largest branch to the latissimus dorsi muscle, and the DIEP pedicle under the microscopic view (Figures 1 and 2) During this process, other small branches to the serratus anterior or latissimus dorsi muscles were excluded.

The Institutional Review Board (Catholic Medical Center Office of Human Research Protection Program) approved our study (IRB approval number: KC19RESI0162).

### 2.1. Measurement of Vascular Diameter

The TD vessels and DIEP pedicle were explored in all patients under loupe magnification (×3.5) as previously reported [21]. The TD vessel was dissected from the bifurcation site with the circumflex scapular artery to the end of each branch that was cut in the immediate proximal part of the next bifurcation, where the vascular diameter decreases by two-thirds as the branching, and the DIEP pedicle was dissected up to just below the bifurcation site of the external iliac artery and vein. After preparing and irrigating with heparinized saline each vessel under the microscopic view, a microvascular approximator clamp was placed on both donor and recipient vessels, and the caliber of each vessel was measured with the microscale ruler in millimeters to the first digit of the decimal point.

### 2.2. Statistical Analysis

Values of vascular diameter were obtained as mean and standard deviation, and *p* values were calculated using repeated measured one-way ANOVA with variables of DIEP vessels and branches of TD vessels. *p* value less than 0.05 indicated a statistically significant difference.

## 3. Results

The baseline characteristics and demographic data of the patients are summarized in Table 1. The average age of the patients was 49.33 ± 7.27, and the average BMI was 23.16 ± 2.54. History of treatment included one radiation, one chemotherapy, two hormone replacement therapies, and eleven previous abdominal surgeries. Others' past histories comprised four hypertensions, one viral hepatitis B, one carotid stenosis, one rheumatoid arthritis, one cerebrovascular accident, one coronary artery disease, and two hypothyroidisms.

The mean diameter of the arterial pedicle of the DIEP flap was 1.88 ± 0.26 mm, and of the venous pedicle of the DIEP flap was 2.03 ± 0.41 mm. Mean arterial diameter was 2.25 ± 0.32 mm for the TD (*p* value < 0.0001), 1.43 ± 0.26 mm for the branch to the serratus anterior muscle (*p* value < 0.0001), 1.70 ± 0.27 mm for the descending branch (*p* value = 0.22), 1.28 ± 0.24 mm for the largest branch to the latissimus dorsi muscle (*p* value < 0.0001), and 1.03 ± 0.15 mm for the second largest branch to the latissimus dorsi muscle (*p* value < 0.0001). Mean venous diameter was 2.52 ± 0.59 mm for the TD (*p* value = 0.0001), 1.83 ± 0.49 mm for the branch to the serratus anterior muscle (*p* value = 0.09), 1.82 ± 0.45 mm for the descending branch (*p* value = 0.07), 1.40 ± 0.33 mm for the largest branch to the latissimus dorsi muscle (*p* value = 0.22), and 1.16 ± 0.24 mm for the second largest branch to the latissimus dorsi muscle (*p* value < 0.0001). Each *p* value was calculated by comparison with the arterial and venous pedicle of DIEP (Table 2).

**Figure 3:**
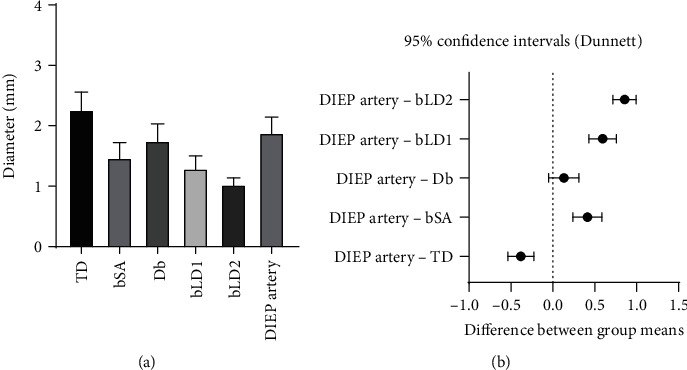
(a) Mean arterial diameters of TD branches and DIEP. (b) 95% confidence intervals using repeated measured one-way ANOVA. The diameter of the DIEP artery was similar to that of the Db; TD: thoracodorsal; bSA: the largest branch to the serratus anterior muscle; Db: the descending branch; bLD1: the largest branch to the latissimus dorsi muscle; bLD2: the second largest branch to the latissimus dorsi muscle; DIEP: deep inferior epigastric perforator.

**Figure 4:**
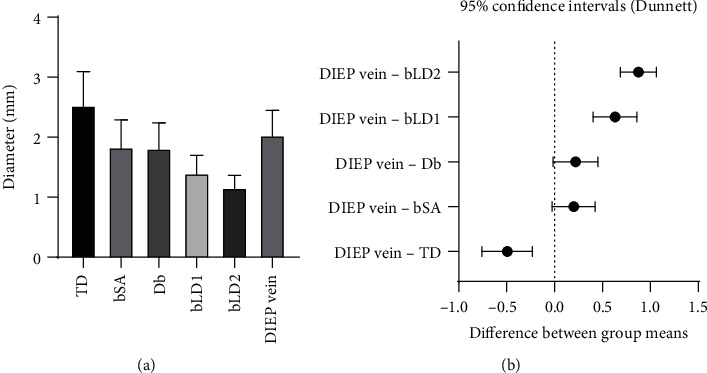
(a) Mean venous diameters of TD branches and DIEP. (b) 95% confidence intervals using repeated measured one-way ANOVA. The diameter of the DIEP vein was similar to those of the bSA and Db; TD: thoracodorsal; bSA: the largest branch to the serratus anterior muscle; Db: the descending branch; bLD1: the largest branch to the latissimus dorsi muscle; bLD2: the second largest branch to the latissimus dorsi muscle; DIEP: deep inferior epigastric perforator.

**Figure 5:**
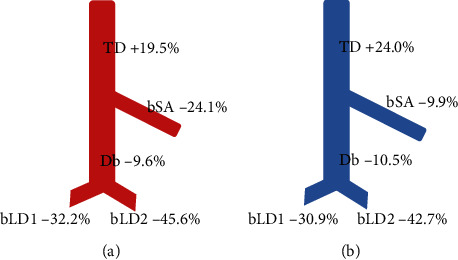
Schema of size differences between the thoracodorsal and the DIEP pedicle expressed as a percentage ((a) artery and (b) vein). Positive numbers mean that TD is bigger, and negative numbers mean that TD is smaller than DIEP. The TD was larger than the DIEP pedicle while the bSA, Db, bLD1, and bLD2 were smaller; TD: thoracodorsal; bSA: the largest branch to the serratus anterior muscle; Db: the descending branch; bLD1: the largest branch to the latissimus dorsi muscle; bLD2: the second largest branch to the latissimus dorsi muscle; DIEP: deep inferior epigastric perforator.

Statistically, the diameter of the DIEP arterial pedicle was similar to that of the descending branch, and the diameter of the DIEP venous pedicle was similar to those of the branch to the serratus anterior muscle and the descending branch (Figures 3 and 4). The TD artery was 19.5% larger than the DIEP arterial pedicle, while the branch to the serratus anterior muscle, the descending branch, the largest branch to the latissimus dorsi muscle, and the second largest branch to the latissimus dorsi muscle were 24.1%, 9.6%, 32.2%, and 45.6% smaller, respectively. The TD vein was 24.0% larger than the DIEP venous pedicle, while the branch to the serratus anterior muscle, the descending branch, the largest branch to the latissimus dorsi muscle, and the second largest branch to the latissimus dorsi muscle were 9.9%, 10.5%, 30.9%, and 42.7% smaller, respectively (Figure 5).

**Figure 6:**
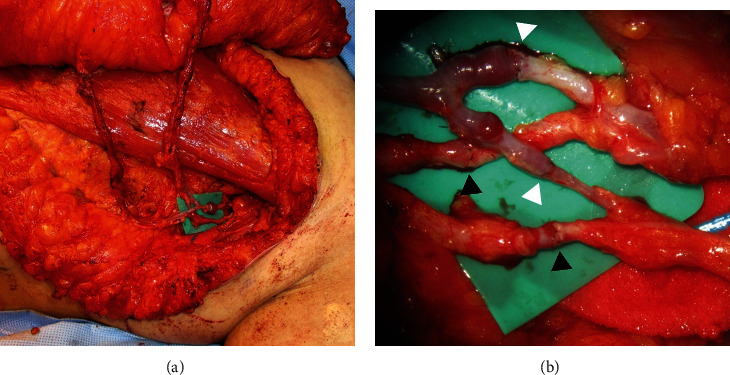
(a) Photo of supercharging anastomosis: both arterial and venous anastomoses were mostly performed with a combination of bSA and Db. (b) Supercharging anastomosis under microscopic view: the black arrowheads indicate the anastomosing sites of arteries, and the white arrowheads indicate the anastomosing sites of veins.

Anastomoses of the artery were indicated for the case needed both in zone I and II to supply the sufficient volume. Those were mostly performed with vessels that showed only a small difference in diameter compared to the DIEP flap, such as the descending branch (56.3%) and branch to the serratus anterior muscle (28.1%). Anastomoses of the vein were performed with the branch to the serratus anterior muscle (29.3%), the descending branch (29.3%), and the largest branch to the latissimus dorsi muscle (24.4%) (Table 3). Four of five arterial supercharged anastomosing cases were fulfilled with the combination of the branch to the serratus anterior muscle and the descending branch. Venous supercharging cases were anastomosed with the branch to the serratus anterior muscle, the descending branch, and the largest branch to the latissimus dorsi muscle (Figure 6 and Supplement Table 1).

In one case, a reoperation was needed to remove the hematoma under the DIEP flap, in which case fat necrosis occurred as the only complication (Table 4).

## 4. Discussion

The results here showed that vessels with nonsignificant difference in size according to ANOVA had increased count of anastomosis, and the difference among the uncoupled vessels was statistically significant. However, this difference is a statistical phenomenon caused by low variation in the standard deviation. When viewed clinically, the uncoupled branches also were recipient vessels, with a metric difference proportion within 30%, and an anastomosis could be performed without size discrepancy even with slight mechanical dilatation. One case of complication was fat necrosis with palpable marginal soft tissue, which did not require a revision. The clinical significance of this study is demonstration of the preparation of TD branches within 30% of the diametric ratio of the DIEP for anastomosis. TD is not only easily approachable and reliable [17, 18] but also has small discrepancy as a recipient for DIEP flap.

For free tissue transfer in breast reconstruction, selection of the recipient vessel is paramount for good microsurgical outcomes [22, 23], and the anastomotic site is the foundation of success in all microvascular procedures [12]. However, size discrepancy of the vessels is a common issue in microvascular anastomosis. Size discrepancy can cause turbulent flow into the flap and is consequently a major risk factor for subsequent thrombotic complications [8–10]. This problem may be pronounced when blood flows from a smaller donor vessel to a larger recipient [24].

Many studies have presented methods to overcome the size discrepancy of the vessels, such as oblique cut anastomosis, fish mouth incision, end-to-side anastomosis, interpositioning graft, and coupling devices [12–16]. However, a minimal size difference can be addressed through judicious dilation by a jeweler forceps [10, 11]. Using a recipient vessel of a suitable size also helps to prevent loss of the free flap in breast reconstruction. If the size difference between the recipient and donor vessels can be predicted, this information can be used to select the appropriate site as a recipient.

Our results showed that the difference in arterial diameter was almost within 30%, when comparing the deep inferior epigastric artery and the TD, branch to the serratus anterior muscle, the descending branch, and the largest branch to the latissimus dorsi muscle. Most anastomoses were performed with the descending branch (56.3%) and the branch to the serratus anterior muscle (28.1%), which showed size discrepancies of 9.6% and 24.1%, respectively.

The branch to the serratus anterior muscle supplies the lower slips of serratus anterior muscle [25] and presents some variation, although the TD anatomy is rather constant [26, 27]. Its normal variation is known to include one (40%), two (50%), or three (10%) branches originating from the TD artery [28]. In cases with two or more branches, the largest vessel was selected for the recipient vessel, in which case no significant differences between vessel diameters were observed according to its positions.

Four of five cases of supercharged anastomosis involved a combination of these two arterial branches. Likewise, the difference in diameter was also less than about 30%, when comparing the deep inferior epigastric vein and the TD, branch to the serratus anterior muscle, the descending branch, and the largest branch to the latissimus dorsi muscle. The venous anastomoses occurred with the branch to the serratus anterior muscle in 29.3% of cases, the descending branch in 29.3% of cases, and the largest branch to the latissimus dorsi muscle in 24.4% of cases.

Arterial second largest branch to the latissimus dorsi muscle showed a 45.6% diametric difference and no case of anastomosis. Venous second largest branch to the latissimus dorsi muscle showed a 42.7% diametric difference, but it was involved in 14.6% of anastomosis cases. The frequency of anastomosis with the largest branch to the latissimus dorsi muscle and the second largest branch to the latissimus dorsi muscle was 38% because it is more advantageous to connect a larger donor pedicle to a smaller recipient vessel [24].

The venous branch with a greater than 40% difference in diameter was used for additional anastomosis, which decreased the occurrence of flap complications. Venous superdrainage is highly effective at increasing the survival of the skin flap [29–31]. It connects the choke vessels and stimulates angiogenesis caused by increased HIF-1*α* and VEGF [29]. Considering that partial necrosis of the DIEP flap is the most common complication related to vein outflow [32, 33], using venous superdrainage can make flap transfer more reliable by altering microcirculation.

Arterial augmentation also shows efficacy for reduced flap necrosis [34–36]. Some animal studies have demonstrated that arterial supercharging is more important for flap viability than venous superdrainage [37, 38]. In flaps with little connection between the vascular territories such as the DIEP flap, arterial augmentation feeding the contralateral side can significantly reduce flap necrosis [39].

In this study, five supercharging anastomoses were observed (Supple. Table 1). One of the five was a delayed breast reconstruction case, and all five showed no complications. The arterial selections consisted of four combinations of branch to the serratus anterior muscle and the descending branch and one combination of branch to the serratus anterior muscle and the largest branch to the latissimus dorsi muscle. Branches of the branch to the serratus anterior muscle and the descending branch had the strongest correlation with the DIEP in diameter. Also, the second selected recipient vessel was considered according to its insetting position. The result shows that a combination of TD branches is sufficient for supercharging anastomosis without complication. The second venous selection was determined by the position of recipient vein. Because the patency of vein was vulnerable, its direction and reachability were the important factors in each selection.

The DIEP flap is considered the most appropriate candidate in autologous breast reconstruction [1–3]. It provides a large volume of well-vascularized autologous tissue, a similar consistency to the natural breast, and esthetic satisfaction, while minimizing morbidity of the abdominal donor site [4–6]. However, large breast reconstruction still remains a challenge for surgeons because of the amount of fat that can be safely transferred with the DIEP flap. The author has overcome this problem by using supercharged anastomosis on a double-pedicled DIEP flap. All four cases of supercharged anastomosis were boosted by arterial augmentation with a combination of the branch to the serratus anterior muscle and the descending branch.

In the vertical inset of the DIEP flap, which is the case with the most frequency, the length between the donor pedicle and the inferior recipient vessel may result in a lack. Thus, the DIEP pedicles have been dissected as far as possible up to just below the bifurcation site of the external iliac artery and vein. Moreover, the cephalic part of flap was rotated to the lateral side to decrease the distance between the perforator site and recipient vessel, because most perforators distribute around the umbilicus in the cephalic part of flap. On the other hand, the turbocharging anastomosis also had to be addressed in order to overcome the shortage of pedicle length in the inferior part. However, in the case of turbocharging, the variation of the diametric difference between flap pedicles was very diverse and technically difficult to anastomose, so the supercharging anastomosis was preferred by the ease to manipulate.

The TD vessels are commonly used recipients for immediate breast reconstructions because they are obtainable after axillary dissection during mastectomy [18]. Although IM vessels have been applied more frequently in recent years, the results of a meta-analysis show that both the IM and TD vessels are safe as recipients without no significant difference in rates of flap failure or other complications [24]. The TD artery is the smaller recipient [17], so it has a more favorable diameter for microsurgical anastomoses. A cadaveric study found that the TD vessels are better recipients of DIEP than the IM and circumflex scapular vessels because they have a smaller size discrepancy [20]. To the best of our knowledge, this is the first clinical study that specifically examined the size difference between the TD vessels and DIEP pedicle in a patient with the microvascular breast reconstruction.

The limitations of the present study are that it is not a randomized controlled trial, but the retrospective design. And the study included a small number of participating patients. Also, interindividual differences may occur when harvesting DIEP flaps and dissecting TD vessels; however, the harvest range and plane were predefined, and the operations were performed in the same manner, to counter this problem.

## 5. Conclusion

Our results suggest good candidates for recipient and supercharging sites of the TD vessel in breast reconstruction with the DIEP free flap. These findings may help prepare the TD vessel with low size discrepancy and reduce the rate of microvascular complications. The descending branch and branch to the serratus anterior muscle showed diametric similarity to the DIEP pedicle and were prioritized for use in anastomosis; these vessels are also expected to be good candidates for supercharging.

## Figures and Tables

**Table 1:** Patient demographics and characteristics.

Variables	Values
Age (yr)	49.33 ± 7.27

BMI	23.16 ± 2.54

Blood pressure (mmHg)	
Systolic	125.85 ± 15.81
Diastolic	75.81 ± 12.70

History of treatment	
Radiation	1 (3.70%)
Chemotherapy	1 (3.70%)
Hormone replacement therapy	2 (7.41%)
Previous abdominal surgery	11 (40.74%)

Past history	
Diabetes mellitus	0 (0.00%)
Hypertension	4 (14.82%)
Others	7 (25.93%)
Smoking	1 (3.70%)

Surgical details	
Delayed reconstruction	2 (7.41%)
Supercharging anastomosis	5 (18.5%)

Values are mean ± SD for continuous variables and number (percentage) for categorical variables. History of abdominal surgery includes one robot-assisted cholecystectomy, two open appendectomies, and eight caesarean sections. Others' past histories comprised one viral hepatitis B, one carotid stenosis, one rheumatoid arthritis, one cerebrovascular accident, one coronary artery disease, and two hypothyroidisms.

**Table 2:** Mean diameters of vessels (mm).

Variable	Values of diameter	*p* value
DIEP		
Artery	1.88 ± 0.26	
Vein	2.03 ± 0.41	

Artery		
TD	2.25 ± 0.32	<0.0001^∗^
bSA	1.43 ± 0.26	<0.0001^∗^
Db	1.70 ± 0.27	0.22
bLD1	1.28 ± 0.24	<0.0001^∗^
bLD2	1.03 ± 0.15	<0.0001^∗^

Vein		
TD	2.52 ± 0.59	0.0001^∗^
bSA	1.83 ± 0.49	0.09
Db	1.82 ± 0.45	0.07
bLD1	1.40 ± 0.33	<0.0001^∗^
bLD2	1.16 ± 0.24	<0.0001^∗^

TD: thoracodorsal; bSA: the largest branch to the serratus anterior muscle; Db: the descending branch; bLD1: the largest branch to the latissimus dorsi muscle; bLD2: the second largest branch to the latissimus dorsi muscle; DIEP: deep inferior epigastric perforator. Values of diameter are mean ± SD, and *p* values are calculated using repeated measured one-way ANOVA with comparison of DIEP vessels and branches of TD vessels. ^∗^*p* value < 0.05 indicates statistically significant difference.

**Table 3:** Frequency and rate of anastomosis with the DIEP pedicle. Values represent numbers (percentages) for categorical variables.

	DIEP artery	DIEP vein	DIEP vein (including venous augmentation)
TD	4 (12.5%)	1 (3.1%)	1 (2.4%)
bSA	9 (28.1%)	10 (31.3%)	12 (29.3%)
Db	18 (56.3%)	11 (34.4%)	12 (29.3%)
bLD1	1 (3.1%)	10 (31.3%)	10 (24.4%)
bLD2	0 (0%)	0 (0%)	6 (14.6%)

Sum	32 (100%)	32 (100%)	41 (100%)

TD: thoracodorsal; bSA: the largest branch to the serratus anterior muscle; Db: the descending branch; bLD1: the largest branch to the latissimus dorsi muscle; bLD2: the second largest branch to the latissimus dorsi muscle; DIEP: deep inferior epigastric perforator.

**Table 4:** Surgical results.

Variables	Values
Complication	
Fat necrosis	1 (3.70%)
Partial flap loss	0 (0.00%)
Complete flap loss	0 (0.00%)

Reoperation	
Hematoma removal	1 (3.70%)
Vascular revision	0 (0.00%)

## Data Availability

The research data used to support the findings of this study are included within the supplementary information file(s).

## Supplementary Materials

Supplementary Materials Supplementary Table 1: information of supercharged anastomosis.
