# Supplementary material for: Diametric Comparison between the Thoracodorsal Vessel and Deep Inferior Epigastric Vessel in Breast Reconstruction
Source: Biomed Res Int. 2020 Jul 16;2020:6352939. doi: 10.1155/2020/6352939 (PMC7381952; doi:10.1155/2020/6352939)
Supplement: Supplementary Materials — Supplementary Table 1: information of supercharged anastomosis. [file 6352939.f1.docx]

| **Patient NO.** | **Age** | **Past history** | **Delayed case** | **Complications** | Artery | | | | | | | | | Vein | | | | | | | | | | |
| --- | --- | --- | --- | --- | --- | --- | --- | --- | --- | --- | --- | --- | --- | --- | --- | --- | --- | --- | --- | --- | --- | --- | --- | --- |
|  |  |  |  |  | TD | bSA | Db | bLD1 | bLD2 | DIEP artery1 | anastomosis | DIEP artery2 | anastomosis | TD | bSA | Db | bLD1 | bLD2 | DIEP vein1 | anastomosis | DIEP vein2 | anastomosis | 3rd vein | anastomosis |
| 1 | 61 | Coronary artery disease | O | no | 2.3 | 1.8 | 1.9 | 1.1 | 1.1 | 2.0 | bSA | 1.8 | Db | 2.2 | 1.8 | 1.8 | 1.4 | 1.0 | 1.8 | bSA | 1.8 | Db |  |  |
| 2 | 38 | Caesarean section | - | no | 2.3 | 1.6 | 2.2 | 1.5 | 1.0 | 2.0 | Db | 1.7 | bSA | 2.1 | 1.4 | 2.0 | 1.8 | 1.4 | 2.0 | bLD1 | 1.6 | bSA | 1.2 | bLD2 |
| 3 | 60 | Robot-assisted cholecystectomy | - | no | 2.3 | 1.6 | 2.2 | 1.5 | 1.0 | 1.5 | Db | 1.5 | bSA | 3.1 | 1.8 | 1.6 | 1.2 | 1.1 | 1.7 | Db | 3.1 | bSA |  |  |
| 4 | 53 | - | - | no | 2.5 | 1.8 | 1.9 | 1.2 | 0.9 | 2.0 | Db | 1.9 | bSA | 3.0 | 2.1 | 2.0 | 1.2 | 1.1 | 2.1 | Db | 2.2 | bSA |  |  |
| 5 | 56 | Caesarean section | - | no | 2.3 | 1.7 | 1.9 | 1.5 | 1.2 | 1.9 | bSA | 1.9 | bLD1 | 2.4 | 2.1 | 1.5 | 1.3 | 1.0 | 2.0 | bSA | 2.1 | bLD1 | 1.2 | bLD2 |
| Mean | 53.60 |  |  |  | 2.34 | 1.70 | 2.02 | 1.36 | 1.04 | 1.88 |  | 1.76 |  | 2.56 | 1.84 | 1.78 | 1.38 | 1.12 | 1.92 |  | 2.16 |  | 1.20 |  |
| SD | 9.29 |  |  |  | 0.09 | 0.10 | 0.19 | 0.19 | 0.11 | 0.22 |  | 0.17 |  | 0.46 | 0.29 | 0.23 | 0.25 | 0.16 | 0.16 |  | 0.58 |  | 0.00 |  |

Supplementary table 1. Information of supercharged anastomosis.
